# Electroadhesion Suction Cups

**DOI:** 10.1002/adma.202420231

**Published:** 2025-04-24

**Authors:** Fabio Caruso, Herbert Shea, Vito Cacucciolo

**Affiliations:** ^1^ Soft Transducers Laboratory (LMTS) École Polytechnique Fédérale de Lausanne Neuchâtel CH‐2002 Switzerland; ^2^ Omnigrasp SRL Bari 70124 Italy; ^3^ RoboPhysics Laboratory (RPL) Politecnico di Bari Bari 70126 Italy

**Keywords:** contract mecahnics, electroadhesion, robotic grasping, soft robotics, suction cup

## Abstract

Suction cups are the light bulbs of robotics and automation. They are simple, reliable, yet energy‐hungry, and require a bulky and noisy vacuum infrastructure. This work reports Electroadhesion (EA) Suction Cups: soft, silent, monolithic, electrically‐driven grippers, with a power consumption of only 1.5 W, that can grasp flat and curved objects, with smooth or rough surfaces, holding payloads up to 1.5 kg. This performance is enabled by a deeper understanding of the contact mechanics of electroadhesion systems. A thin and soft membrane containing interdigitated electrodes zips onto the object driven by electrostatic forces, conforming to the object's shape and thus establishing large‐area contact. The lifting force is transmitted to a robot arm through a small pillar connected at the center of the membrane. This design maximizes the peeling force and enables the formation of passive vacuum inside the conical chamber formed when the membrane stretches during lifting. Object release is obtained by turning off the voltage and optionally by opening a valve to quickly break the vacuum. EA suction cups address many shortcomings of widely used vacuum‐driven grippers, offering a compact, fully electric, and energy‐efficient solution that meets the needs for efficiency and portability in both industrial and service robotics.

## Graphical abstract

Electroadhesion (EA) Suction Cups are electrically operated soft grippers that can pick up a wide range of objects from the top. A soft membrane, driven by electrostatic forces, conforms to surfaces, creating a passive vacuum when lifted. EA suction cups eliminate bulky vacuum systems, offering a compact, efficient, and portable alternative for industrial and service robotics.

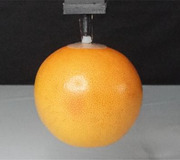

## 1 Introduction

Despite the wide body of research on robotic grippers, both soft and rigid^[^ ^1^ ^]^ grasping from the top is still largely done using suction cups, a >100 year‐old technology.^[^ ^2^ ^]^ Grasping from the top is required whenever objects are closely packed, a common case in industry (e.g., boxing, bin picking), in retail stores, and in home environments.

Industrial suction cups use active vacuum: the required flow of air is generated by a vacuum pump or converted from compressed air using a venturi ejector. The reasons for the enduring success of active suction cups lie in their simple rugged design, versatility, and straightforward control. They can easily pick objects of different materials and shapes, from flat to curved, with smooth to rough surfaces.^[^ ^3^ ^]^ However, the infrastructure needed to generate vacuum is noisy, bulky (vacuum generator, tubes, manifolds, valves), and energy hungry.^[^ ^4^ ^]^ Power savings are becoming a top priority in many industries. There is a growing need for compact, portable, and low‐power robotic end‐effectors.

Researchers have developed alternative solutions for grasping objects from the top that do not use active vacuum. Yet these solutions tend to address niche applications or objects with specific properties. A non‐vacuum gripper that can grasp objects from the top, combining the simplicity and versatility of suction cups, has not been reported to date. Robotic hands show high versatility, but they need to grasp objects from the sides and tend to be more complex and expensive than suction cups.^[^ ^5^ ^]^ Passive suction cups, for example, do not need active vacuum, but their use is limited to flat, rigid, and very smooth surfaces due to the inability to conform and maintain vacuum on curved and rough objects. They also require a pre‐load to generate vacuum, which prevents their application on soft and deformable objects.^[^ ^6^ ^]^ Gecko‐adhesive grippers^[^ ^7^ ^]^ are low‐power since they create adhesion using Van der Waals forces between soft micropillars and the target objects.^[^ ^8^ ^]^ Their adhesion forces are strongly influenced by surface roughness,^[^ ^9^ ^]^ due to the short range of action of Van der Waals forces. Object release is also challenging and it usually requires an active bending or stretching of the gripper.^[^ ^10^ ^]^ Octopus‐inspired suction cups use a thin, stretchable membrane covering the edge of the suction cup.^[^ ^11^, ^12^, ^13^ ^]^ These grippers tend to be more delicate and consume less energy than conventional suction cups. The membrane helps also in separating the fluidic components from the environment enabling underwater applications.^[^ ^14^ ^]^

Electroadhesion (EA) grippers grasp delicate objects with very low power consumption using electrically‐induced adhesive forces. They offer many advantages in terms of simple control, silent operation, adaptation to curved shapes, and smooth to rough surfaces. The phenomenon of electroadhesion was discovered more than one century ago^[^ ^15^ ^]^ and several grippers based on EA have been reported since then.^[^ ^16^ ^]^ Rigid EA systems are widely used in the semiconductor industry to automate the handling of silicon wafers,^[^ ^17^ ^]^ and in the textile industry to handle fabrics.^[^ ^18^ ^]^ Electroadhesion was also explored in other applications including flying robots perching^[^ ^19^ ^]^ and soft wall‐climbing robots.^[^ ^20^ ^]^ A summary of the technologies and details of their applications can be found in review articles.^[^ ^21^, ^22^ ^]^

Recent studies on electroadhesion have shown that a deeper understanding of contact mechanics^[^ ^23^ ^]^ and charge accumulation at the interface between different dielectrics,^[^ ^24^ ^]^ can significantly improve device performance, resulting in increased force per unit area and reduced power consumption. Kim et. al. in 2020^[^ ^25^ ^]^ reported electroadhesive clutches that operate in the 1 V range using ionic elastomers that create charge separation across the molecular‐scale ionic double layer. These findings represent fundamental breakthroughs that tackle some of the limitations of current electroadhesion technologies, paving the way for broader applications.

EA grippers made of stretchable and flexible materials^[^ ^26^ ^]^ offer much higher versatility and holding forces than their rigid counterparts. Soft EA grippers can conform to a wide variety of shapes and sizes creating a sufficiently large contact area that allows them to grasp delicate objects with a wide range of sizes and shapes, holding loads over 1000 times their weight.^[^ ^27^ ^]^

However, the grasping force of soft EA grippers depends very strongly on the grasping posture. This phenomenon is due to the dependence of the EA force on the peeling angle between the EA gripper and the object surface.^[^ ^28^ ^]^ The peeling behavior of EA fingers is very similar to the one of adhesive tapes attached to a flat surface: the holding force is very high when the tape is pulled parallel to the surface and very low when the tape is pulled perpendicular to it. It follows that EA soft grippers can lift kilograms when grasping objects from the sides or when wrapping their fingers below the object, while their holding force reduces to a few millinewtons when lifting objects from the top.^[^ ^29^, ^30^ ^]^ This limitation is a key element that held back the widespread industrial adoption of soft EA grippers.

To tackle this challenge, Piskarev et al.^[^ ^31^ ^]^ developed a gripper combining granular jamming with EA, mitigating the effects of EA peeling when grasping objects from the top thanks to variable stiffness (the gripper is soft when contacting the object and rigid when lifting it). Okuno et al.^[^ ^32^ ^]^ integrated an EA soft pad at the edges of an active suction cup to reduce air leakage on rough surfaces. Both papers report improved performance in grasping objects from the top. Yet, they both require a vacuum infrastructure.

**Figure 1:**
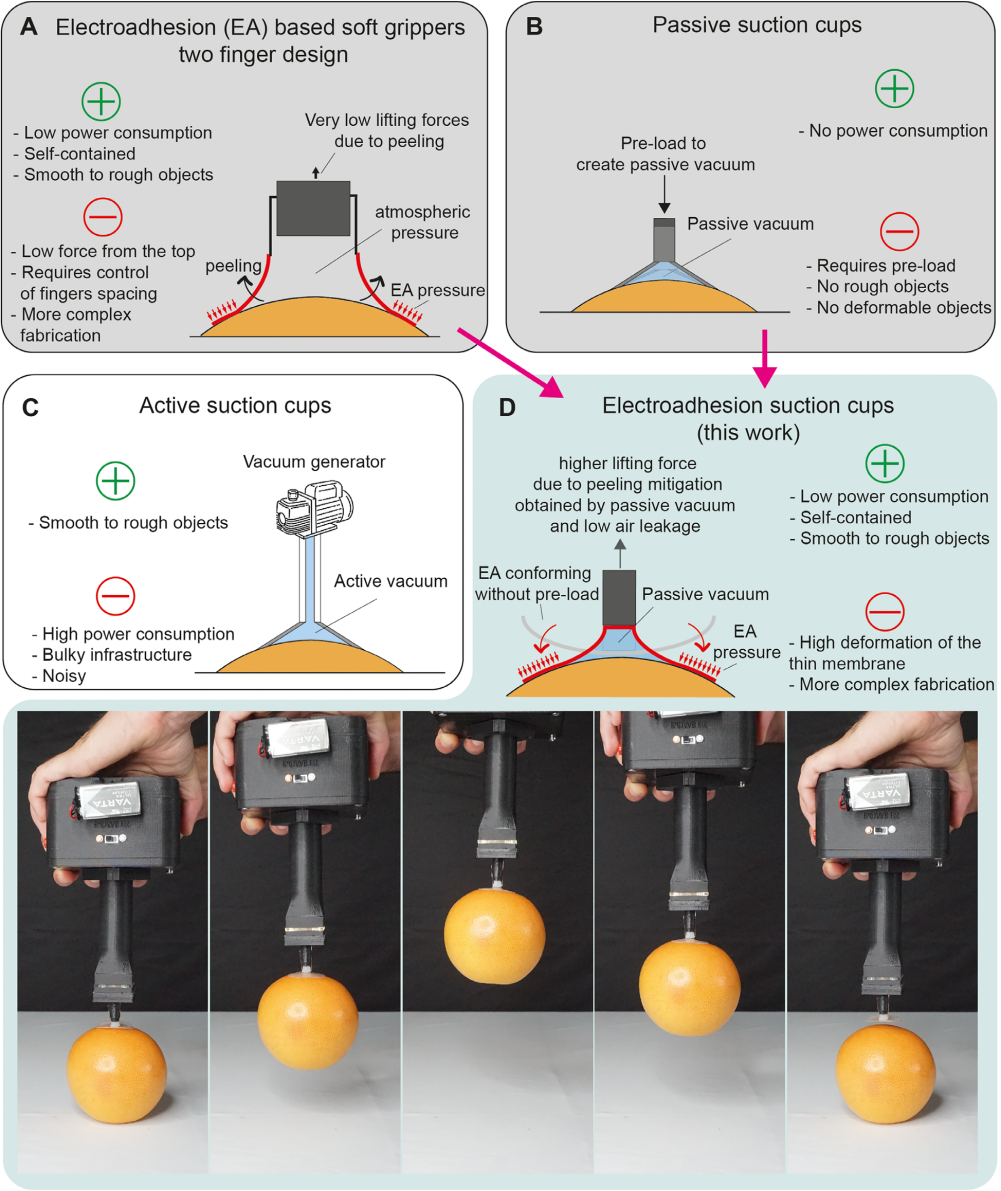
Comparison of grasping mechanisms that inspired the design of the electroadhesion suction cups. A) Electroadhesion (EA) soft grippers with two‐finger design, characterized by low power consumption and the ability to handle smooth to rough objects, but limited by low lifting forces from the top due to peeling. B) Passive suction cups do not need active vacuum (e.g., a pump), but their use is limited to flat, rigid, and very smooth surfaces due to the inability to conform and maintain vacuum on curved and rough objects. C) Active suction cups are widely used thanks to their simple design, versatility, and simple control. They can easily pick objects of different materials and shapes, from flat to curved, with smooth to rough surfaces. However, they require a vacuum infrastructure (vacuum generator, tubes, manifolds, valves) and consume significant power. D) EA suction cups, reported in this paper, can grasp heavy objects from the top without any vacuum system. The key novelty, essential to reach lifting forces in the order of kilograms, is the interplay between EA and the passive vacuum that forms when the EA membrane deforms.

We present here an EA gripper design that can grasp heavy objects from the top without any vacuum system. The key design element that allows reaching holding forces of kilograms is the interplay between EA and the passive vacuum that forms when the EA membrane deforms as the gripper is lifted. We named these grippers EA suction cups, because they combine the advantages of EA grippers (low power, fully electrical, self‐contained), with the ones of passive suction cups (large forces from the top), as displayed in **Figure** **1**. The EA suction cups can grasp both flat and curved objects, with smooth to moderately rough surfaces (Root mean square roughness Sq = 1.9 µm), with a payload >1.5 kg, with no preload, and with pick and release time <0.4 s. Fast release is achieved using a miniaturized solenoid valve to vent the passive vacuum.

**Figure 2:**
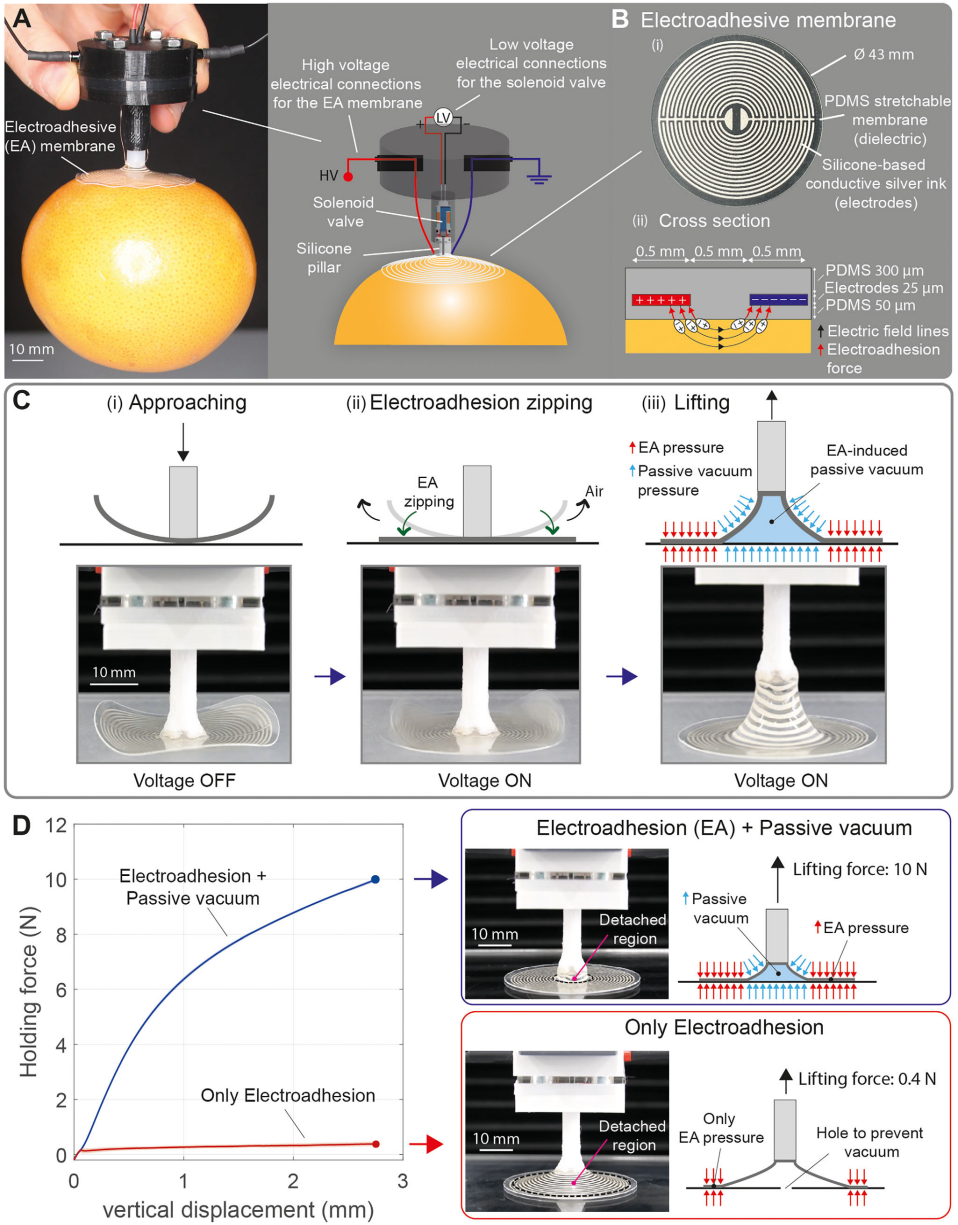
A soft electroadhesive suction cup. A) Electroadhesive (EA) soft suction cup grasping a delicate object (grapefruit) without damaging it. This gripper can grasp objects from the top, is fully electrical and self‐contained (no vacuum hose required), and features very low steady‐state power consumption (1.5 W). The gripper consists of a soft EA membrane attached to a soft 3D‐printed pillar made of Thermoplastic Elastomer (TPE). A solenoid valve is integrated inside the pillar to allow quick venting for rapid object release. B) The structure of the electroadhesive membrane: i) geometry of the interdigitated stretchable electrodes, ii) schematic cross‐section. C) EA suction cup operating principle. i) The gripper approaches the object at zero voltage. ii) When in contact with the object, the voltage is applied (3 kV DC), causing the EA membrane to conform (EA zipping) to the object surface. iii) The object is then lifted, causing the deformation of the thin membrane, starting from the center. This deformation creates a conical chamber. Due to the airtight sealing enabled by the EA pressure, passive vacuum is created inside the conical chamber. This version of the gripper does not have a valve, and the electrical connections are integrated inside the pillar. D) Lifting force as a function of vertical displacement with and without a small hole (0.2 mm diameter) in the PMMA substrate, illustrating a significant improvement in lifting performance with the EA‐induced passive vacuum. Values are mean ± SD (shaded area) from N = 3 trials.

This unprecedented performance is enabled by a design guided by a better understanding of contact mechanics for electroadhesion systems. The gripper is made of a thin and soft circular membrane with embedded interdigitated electrodes, which generate EA forces (**Figure** **2**A,B). The membrane is connected to the gripper frame only at the center using a small pillar. A miniaturized valve is integrated into the pillar to vent passive vacuum to enable very fast object release. We carefully designed how the EA membrane deforms when lifting the object.

The combination of three key effects (large peeling boundary, low peeling angle, and passive vacuum) allows the EA suction cups to obtain a 10^4^ increase in lifting force from the top compared to EA grippers with a two‐finger design^[^ ^27^ ^]^ (Figure 2D).

The sequence for lifting is a robot arm positions the center of the EA membrane in contact with the object to be picked up. The voltage is applied, and the electrostatic forces deform the membrane, making it zip conformally on the object. Thanks to its free boundaries, the EA membrane can easily deform, adapting to the shape of the object, and establishing large‐area contact.^[^ ^29^ ^]^ The robot arm then raises the gripper, and the EA membrane starts detaching from the object at the center, where the lifting force is transmitted through the pillar. A circular peeling boundary is created around the connection with the pillar. The EA peeling force is proportional to the length of the peeling boundary and inversely proportional to the peeling angle.^[^ ^28^ ^]^ In our design, the peeling boundary takes the shape of a circle whose radius increases with the load, and the peeling angle is constrained mechanically and kept at a low value.^[^ ^33^ ^]^ The combination of a large peeling boundary and a low peeling angle dramatically increases the peeling force of EA suction cups compared to a two‐fingered EA gripper grasping objects from the top (0.4 N compared to 5 mN).

When lifting the gripper, an EA‐induced vacuum chamber is formed below the pillar due to the EA membrane partial detachment and stretching. This vacuum chamber is kept sealed by the EA pressure between the outer portion of the membrane and the object. The vacuum pressure keeps the object attached to the EA membrane, further increasing its lifting force.

We demonstrate the use of one EA suction cup to pick and place a wide range of objects: rigid and deformable, with materials ranging from plastic to food to metals, shapes from flat to curved, and sizes ranging from 5 to 20 cm (Movie S1, Supporting Information). The gripper is driven by a compact power supply (10 cm on a side) requiring only a 12 V DC input and on/off digital signals. EA suction cups can bring dramatic advantages in robotic grasping, offering a highly integrated gripper solution that can grasp a wide variety of objects with very low noise and power consumption of 1.5 W. A comparison with previously reported soft gripper capable of grasping from the top is given in **Table** **1**.

**Table 1:** Performance comparison of soft grippers capable of grasping objects from the top.

Gripper type	Technology	Maximum weight lifted per gripper area [N/cm^2]	Requires Preload	Pick time/ Release time [s]	Power consumption [W]	Object shape: (B) Both flat and curved (C) Only Curved	Surface conditions:(A) Any, (D) Dry and clean	Requires vacuum infrastructure
Standard vacuum suction cups^[^ ^4^ ^]^	Active vacuum	5.5	No	0.2 / 0.02	4.5 W – 68 W^b)^	(B)	(A)	Yes
Adaptive self‐sealing suction‐based soft gripper^[^ ^11^ ^]^	Active vacuum + thin self‐sealing membrane	1.1	Yes	8 / 2	–	(B)	(D)	Yes
Magnetically switchable soft suction grippers^[^ ^34^ ^]^	Passive vacuum + Magnetorheological fluid	2.4	Yes	10 / ‐	–	(B)	(A)	No
Universal gripper based on jamming of granular materials**^[^** ^35^ ^]^	Granular jamming	1	Yes	–	–	(C)	(A)	Yes
Magnetorheological fluid‐based robotic end effector^[^ ^36^ ^]^	Granular jamming + Magnetorheological fluid	0.8	Yes	0.1 / ‐	75 W	(C)	(A)	No
Stretchable Suction Cup with Electroadhesion^[^ ^32^ ^]^	Electroadhesion + Active vacuum	2.9	No	–	–	(B)	(A)	Yes
Soft gripper with Granular jamming and Electroadhesive properties^[^ ^31^ ^]^	Electroadhesion + Granular jamming	1.2	Yes	0.2 / 0.2	–	(B)	(A)	Yes
Shear adhesion gripper with fibrillar thin film^[^ ^37^ ^]^	Gecko adhesion (Van der Waals forces)	1.2	No	0.1 / 0.2	^a)^	(C)	(D)	No
**Electroadhesion Suction cups (This work)**	**Passive vacuum + Electroadhesion**	0.8	**No**	0.2 **/** 0.1	**1.5 W**	**(B)**	**(D)**	**No**

^a)^ Power required to release the objects;

^b)^ See Section 2.6 and Supporting Information for further details.

## 2 Results

### 2.1 Working principle

The gripper operates using a combination of electroadhesion (EA) and passive vacuum induced by mechanical deformation of the stretchable membrane. A gripper device includes (Figure 2A): 1) an EA soft membrane that contacts the object, 2) a central pillar that connects the EA membrane to the gripper case, 3) a miniaturized solenoid valve coaxially mounted in the pillar, 4) a case containing electrical wiring and enabling the mechanical connection to a robot arm. The EA soft membrane is a circular (4.3 cm in diameter) silicone film with embedded stretchable electrodes with an interdigitated circular design (Figure 2B(i)). The electrodes are connected to a 3 kV power supply. The interdigitated electrodes have an in‐plane spacing of 0.5 mm and are separated from the object by a 0.05 mm‐thick dielectric silicone film. When a voltage is applied between these electrodes, fringing electric fields polarize the surface of the object, creating electrical adhesion.

The grasping process includes the following steps (Figure 2C): i) the gripper is brought into contact with the object (the pillar region touches), ii) the voltage is turned on and the EA thin membrane conforms to the shape of the object, zipping from the center toward the outside, pushing the air out and creating a strong seal with the object, iii) the gripper is lifted, transferring the lifting force from the pillar to the EA membrane, which deforms in a conical shape, creating a volume filled with vacuum, which balances the weight of the object together with the EA forces.

The EA membrane needs to be thin, stretchable, and connected to the body of the gripper only at the center. For the electrostatic zipping to happen, the change in electrostatic energy (that depends on the voltage and capacitance of the stretchable electrodes) must overcome the change in mechanical energy (membrane stiffness) from the unzipped configuration (at rest) to the zipped one.^[^ ^29^ ^]^ We designed the EA membrane as a thin (300 µm thickness) and soft (Silicone with elastic modulus = 3.9 MPa) membrane, only attached at the central pillar. This geometry ensures that during zipping the air is evacuated and a large contact area is created, without air bubbles or open channels. We controlled the curing temperature of the top silicone layer to generate residual stresses that bend the membrane upward, ensuring only the center initially contacts the object, and air is pushed out from the center outward during EA zipping.

During lifting, the EA membrane deforms with a conical shape due to the lifting force applied by the pillar to the center of the membrane. The deformed membrane creates a vacuum chamber whose volume depends on the applied force, membrane stiffness, and object roughness.

To measure separately the force due to passive vacuum and the EA peeling force, we tested our gripper on a PMMA substrate with a 0.2 mm hole (Figure 2D). The hole in the substrate connects the deformed chamber in the EA membrane to atmospheric air, preventing vacuum formation during lifting. Tests were conducted using an EA membrane (4.3 cm in diameter) with an applied voltage of 3 kV DC. Without the hole (EA peeling plus passive vacuum), the gripper produced a grasping force of 10 N at a vertical displacement of about 3 mm. With the hole (i.e., with only EA peeling force as no vacuum could be generated), the force reached 0.4 N at detachment. This result confirms our previous finding that EA force is strongly influenced by the peeling effect, so even on smooth surfaces and with good contact, when lifting from the top (initial peeling angle 90°) the holding force is orders of magnitude lower than when lifting from the sides (peeling angle 0°).^[^ ^28^ ^]^ With respect to our previous article on EA peeling, we were able here to increase the EA force for vertical lifting thanks for the axially symmetric design that decreases the peeling angle due to constraints imposed by the membrane deformation, reaching a pure EA lifting force of 0.4 N with a 20 mm radius membrane, compared to 5 mN for a 20 mm wide tape. This pure EA force is then further increased by over 20x thanks to passive vacuum.

To quantify the relative contribution of passive vacuum on the lifting force, we measured the pressure inside the deformed chamber during lifting using a vacuum pressure sensor placed below the testing plate. The experimental setup and results are shown in Figures S3 and S4 (Supporting Information). The experimental procedure is described in Experimental Section. The data show a very good agreement between the force measured directly by the load cell and the force determined from the product of vacuum pressure and detached area (Figure S4A, Supporting Information). These results show that when passive vacuum forms well, the lifting force is almost entirely due to the vacuum. The contribution of the electroadhesion pressure on the lifting force is negligible. The role of the electroadhesion is, however, crucial to ensure good sealing, which is necessary for passive vacuum to form.

### 2.2 Influence of Substrate Roughness

The passive vacuum relies on the strong seal between membrane and object provided by EA pressure. The amount of air leaking into the vacuum cavity depends on: 1) the EA pressure, 2) the membrane material, and 3) object roughness. Traditional passive suction cups made of elastomers will detach from the wall or glass after some time, due to continuous air leakage through the seal.^[^ ^38^ ^]^

Predicting such air leakage is complex since surface roughness spans a wide range of length scales, from nanometers to millimeters. Studies based on multi‐scale contact mechanics coupled with fluid dynamics show that air leakage depends mainly on the membrane‐substrate contact pressure and the stiffness of the membrane, for a given roughness power spectrum.^[^ ^39^ ^]^

**Figure 3:**
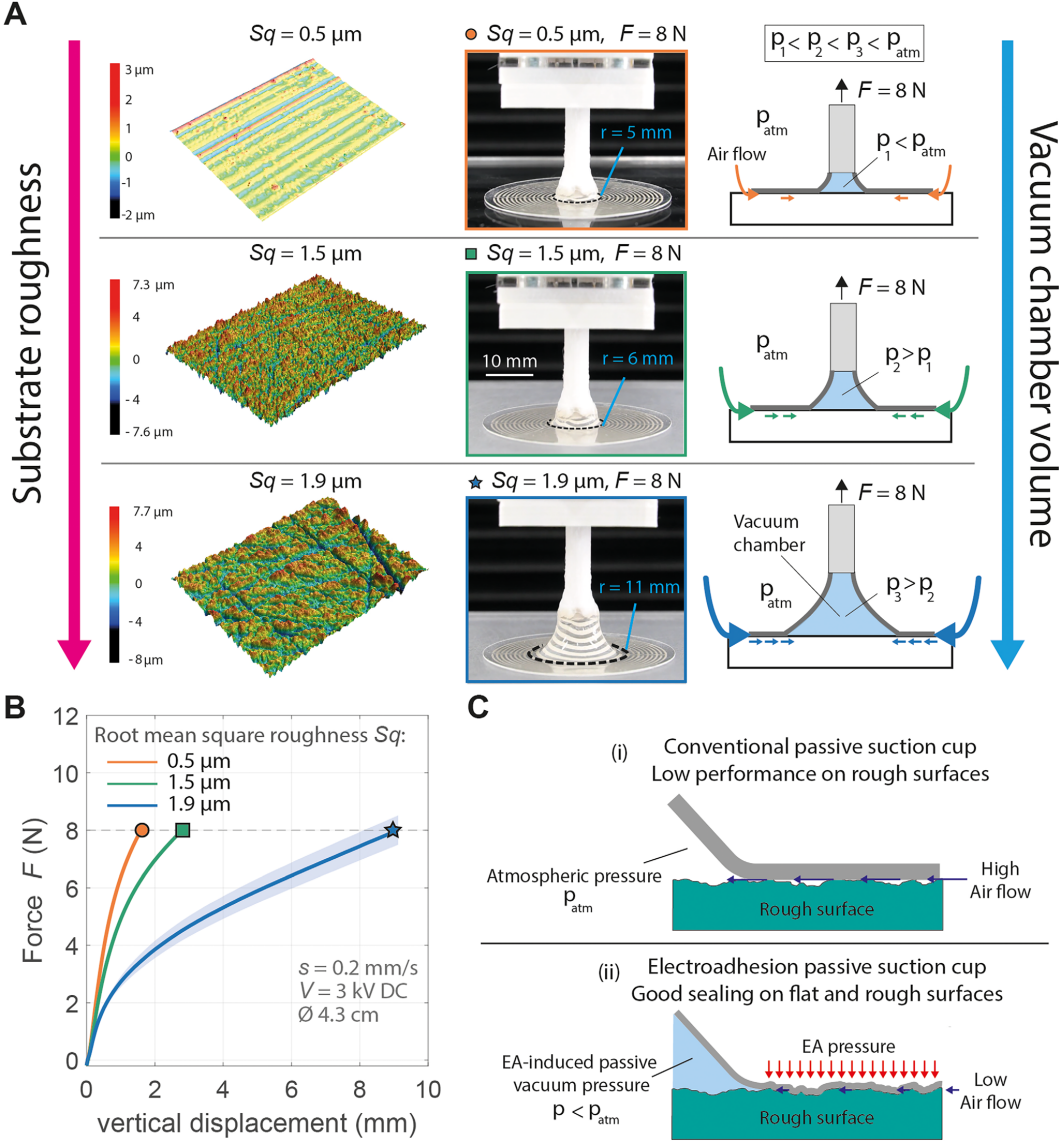
Influence of substrate roughness on lifting performance. A) From top to bottom, the roughness is increased. During lifting, air leakage occurs more strongly for high substrate roughness, resulting in higher vacuum pressure inside the deformed chamber, thus requiring larger deformation to reach the same lifting force. From left to right: Topographical 3D images of the 3 PMMA substrates, used in the tests, extracted from the confocal microscope. Pictures of the EA suction cups at 8N of lifting force with three different vacuum chamber volumes (*r* indicates the radius of the detached area). Schematic cross‐section of the EA suction cup showing air leakage path, vacuum pressure, and chamber deformation. B) Lifting force as a function of vertical displacement for PMMA rigid substrates with different surface roughness (measured using root mean square height Sq). As the roughness of the substrate increases, air leakage increases, resulting in lower vacuum pressure inside the deformed chamber, which means that a larger deformation is needed to reach a given lifting force. All tests were conducted at a vertical speed of s = 0.2 mm s^−1^. The plots show mean ± SD (shaded area) for N = 3 trials for each substrate roughness. C(i)) Conventional suction cups fail to create effective sealing on rough surfaces. ii) Thanks to a thin and soft membrane design and the Electroadhesion pressure, EA suction cups can create effective sealing also on moderately rough substrates.

We decided to estimate the air leakage through the EA membrane seal experimentally. We tested three PMMA substrates (**Figure** **3**A): one smooth without any treatment (Root mean square roughness Sq = 0.5 µm), one polished with sandpaper with grit size 320J (Sq = 1.5 µm), and one with grit size 80J (Sq = 1.9 µm). In each test, an EA membrane (diameter 4.3 cm) attached to a rigid pillar is adhered by EA to the substrate and pulled vertically at a constant speed (s = 0.2 mm s^−1^) measuring the lifting force (more details in the Experimental Section and Figure S1, Supporting Information). Each test stops when a lifting force of 8 N is achieved, to avoid damaging the membrane.

During lifting, more air leakage occurs for substrates with higher roughness. We measured the radius of the passive vacuum chamber at 8 N load for substrates with three different roughness values. If full vacuum were present inside the deformed chamber (i.e., no air leakage) the radius of the detached area would be *r*  =  5 mm at a lifting force of 8 N. This value of detached radius is obtained using the formula *F*  =   − *p_v_* π*r* ^2^, with *p_v_* = ‐101 kPa, the gauge pressure in the vacuum chamber. For the experiments conducted on smooth PMMA substrates (Sq = 0.5 µm), the detached radius at 8 N is ≈5 mm, indicating that for smooth surfaces the EA pressure ensures a very effective sealing. A gauge pressure close to ‐101 kPa can be easily reached with small vertical deformation (< 2 mm). As the roughness of the substrates increases, air leakage increases, resulting in higher pressure inside the deformed chamber. Therefore, a larger deformation is required to obtain a given lifting force (r = 6 mm for Sq = 1.5 µm and r = 11 mm for Sq = 1.9 µm, see Figure 3A).

The results are summarized in Figure 3B. The slow vertical speed used in these tests was deliberately chosen to clearly visualize the influence of roughness on air leakage. Because air leakage is a time‐dependent phenomenon, the curves plotted in Figure 3B would be very similar to each other at high lifting speeds.

These tests demonstrate that the EA suction cups presented in this work are effective also on moderately rough substrates (Sq = 1.9 µm), reaching forces >8N, thanks to the good sealing provided by the very thin and soft EA membrane under the EA pressure. By contrast, traditional passive suction cups only work on very smooth surfaces. Passive suction cups rely on the elastic restoring force of their body to create passive vacuum, so they must have substantial stiffness, which prevents sealing on rough surfaces Figure 3C.

### 2.3 Influence of Membrane Diameter, Material Stiffness, and Applied Voltage

We investigated the effect of membrane diameter, material stiffness, and applied voltage using the experimental setup shown in Figure S1 (Supporting Information). We fabricated two types of EA suction cups using either 1) a soft and stretchable bottom layer made of PDMS (thickness 50 µm, elastic modulus 3.9 MPa) or 2) a stiff and flexible bottom layer made of Mylar (thickness 23 µm, elastic modulus 4.1 GPa). In both cases, we used a 300 µm thick PDMS layer to cover the electrodes (fabrication process described in Experimental Section).

**Figure 4:**
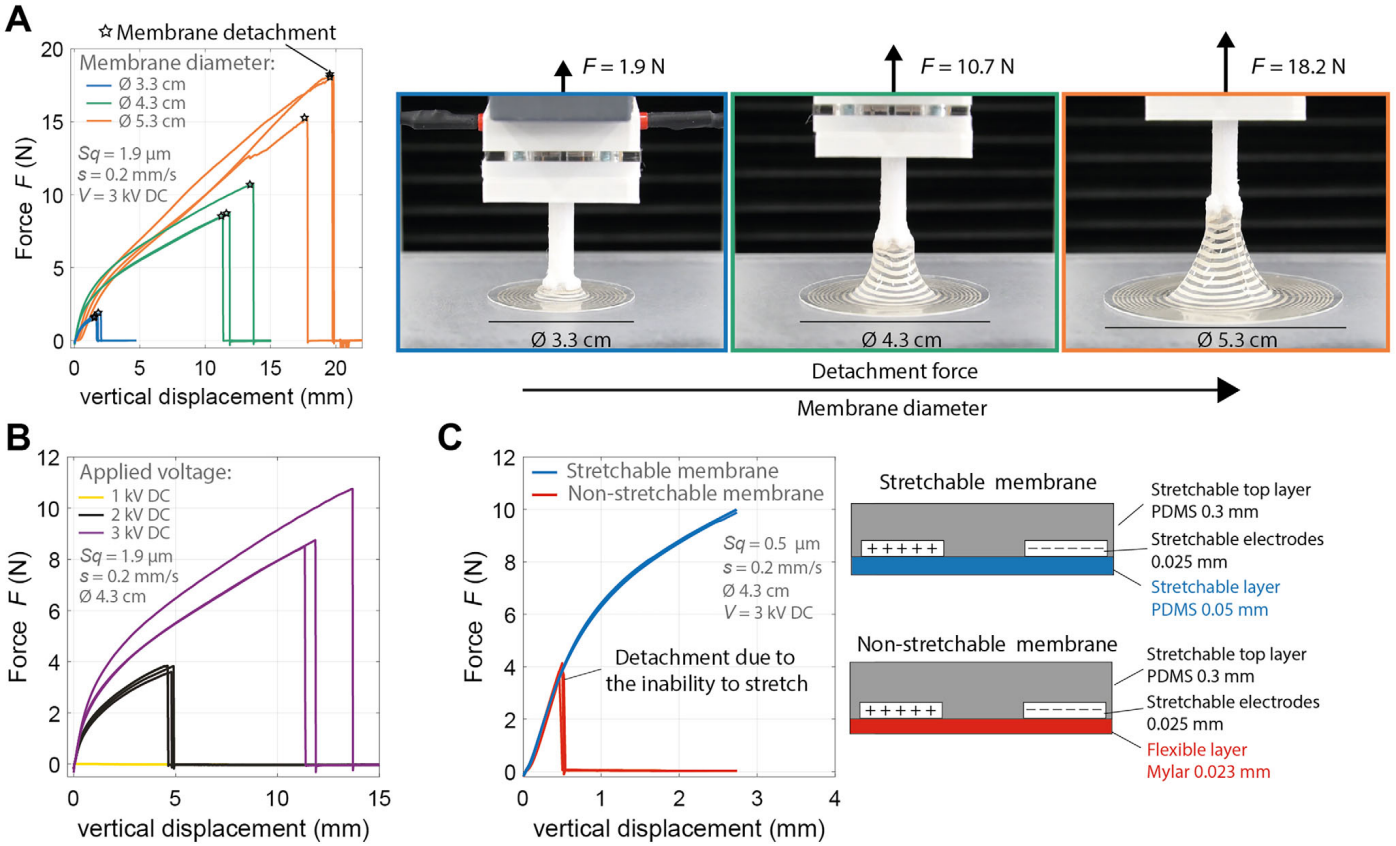
Influence of membrane diameter, applied voltage, and membrane stiffness on lifting force. A) Lifting force during vertical displacement for different membrane diameters. All the tests were conducted on a PMMA substrate of roughness *Sq* = 1.9 at a vertical speed of s = 0.2 mm s^−1^ and an applied DC voltage *V* = 3 kV. N = 3 trials were conducted for each membrane diameter. B) Lifting force during vertical displacement varying the applied voltage and keeping constant the membrane diameter. C) Lifting force during vertical displacement for two membranes with different material stiffness. During the initial 0.5 mm of lifting displacement, the two membranes show the same trend. The flexible membrane (red curves) detaches earlier than the stretchable membrane (blue curves) due to the inability to stretch.

We tested the effect of membrane diameter using stretchable EA suction cups with diameters 3.3, 4.3, and 5.3 cm, and we measured the lifting force as a function of vertical displacement on a rough surface with *Sq* = 1.9 µm and using a vertical velocity of 0.2 mm s^−1^ (**Figure** **4**A). As expected, larger diameters lead to higher detachment forces. Detachment happens before the vacuum chamber reaches the edge of the membrane, due to membrane buckling and slipping in the adhered region. Figure 4A shows pictures of the three membranes at the instant when detachment happens. The detachment force depends on three main factors: 1) the area of the detached vacuum chamber, 2) the length of the peeling edge,^[^ ^33^, ^40^ ^]^ and 3) the EA sealing area, which is inversely proportional to the air leakage (larger area leads to lower leakage, hence to higher vacuum pressure in the vacuum chamber).

We then characterized the effect of the applied voltage on the lifting performance, as shown in Figure 4B. We used a stretchable membrane with a diameter of 4.3 cm. For an applied voltage of 1 kV the EA energy is too low for this membrane to start zipping on the substrate, a rough PMMA plate with *Sq* = 1.9 µm. No lifting force is generated. At 2 kV and higher, the membrane rapidly zips on the substrate. Once zipped, the EA pressure creates the air seal. The EA pressure is due to the Maxwell stress which is, to a first approximation, proportional to the square of the electric field in the direction perpendicular to the contact surface. Therefore, a higher voltage leads to higher EA pressure, which leads to a higher detachment force, due to both lower air leakage (lower vacuum pressure) and higher peeling force.

Finally, we characterized the influence of the membrane material stiffness. Figure 4C plots the measured lifting force as a function of vertical displacement. Until a vertical displacement of 0.5 mm, the behavior of both stretchable (PDMS) and flexible (Mylar) EA suction cups is very similar. For very low vertical displacement the flexible gripper can deform without stretching. For higher displacements, the flexible membrane cannot move vertically without in‐plane stretching, leading to buckling and a drop in the holding force (open channels form, allowing air into the vacuum cavity). By contrast, the stretchable membrane can deform without buckling, keeping the air seal and reaching higher forces at higher vertical displacements.

Membrane thickness and material stiffness both strongly influence the performance of EA suction cups. In this article we selected materials from validated EA grippers in the literature^[^ ^28^, ^29^ ^]^ and compatible with fabrication processes we have access to. Material properties and geometry influence EA zipping performance, EA pressure, membrane deformation, air leakage through the membrane. These parameters are coupled together and form a complex design space. Future work will be conducted to explore such design space through a physical model and comparing membranes made with different materials and geometries.

### 2.4 Holding and Release Time

We then characterized the holding and release time of the EA suction cups lifting a constant load. The goals are: 1) to measure how long a gripper can hold an object and 2) how fast the gripper can release it. The experimental setup is shown in Figure S2 (Supporting Information). The substrate is a 720 g PMMA box, free to move vertically. In a first experiment, we measured the maximum holding time as a function of substrate roughness, which influences the air leakage in the EA sealing (see section “Influence of substrate roughness”). We used the same surface roughness values tested in the previous section (same process: sanding the PMMA with sandpaper of different grit sizes). The substrate is lifted by 12 mm while the voltage is kept on. **Table** **2** first column reports the holding time.

**Table 2:** Maximum holding time and release time comparison lifting a constant load (720 g) with different substrate roughness.

Substrate Roughness Sq [µm]	Maximum Holding time [s] Voltage ON No valve	Release time [s] Voltage OFF + Valve Closed	Release time [s] Voltage OFF + Valve Open
**0.5**	>300	>300	<0.153 ± 0.04
**1.5**	149.9 ± 4.7	85.3 ± 3.5	<0.125 ± 0.04
**1.9**	51±1.7	19.9 ± 0.8	<0.083 ± 0.04

The substrate roughness affects significantly the holding time. For smooth surfaces (Sq = 0.5 µm), we measured a holding time higher than 300 s. We stopped the experiments at 300 s for practical reasons, since we are interested in pick and place applications, which usually last only a few seconds. As the substrate roughness increases, air leakage increases as well, leading to lower holding times. For moderately rough surfaces (Sq = 1.9 µm) holding times are ≈ 50 s. These results show that EA suction cups can be used for pick and place applications of smooth to moderately rough objects with weights over 500 g with holding times of tens of seconds, well beyond the typical duration of pick and place cycles (1–5 s).

We further characterized how the load influences the holding time using a PMMA substrate with roughness (Sq = 1.5 µm). By varying the load from 100 to 700 g we measured holding times from 720 to 120 s (Figure S5, Supporting Information). As expected, heavier objects lead to lower holding times, since higher weights produce lower pressure inside the deformed chamber. This higher pressure difference between the deformed chamber and the atmosphere leads to higher airflow through the electroadhesion seal, in turn increasing the pressure in the deformed chamber in a shorter time.

We then characterized the release time, which is essential for pick and place applications. It is well‐known that EA grippers can suffer from slow release, due to both residual charges and dry adhesion. Table 2, second column, reports release times on the same objects (720 g PMMA box, roughness *Sq* from 0.5 to 1.9 µm) when voltage is removed but the integrated venting valve is kept closed. We used a bipolar 3 kV AC voltage at 5 Hz to reduce residual charges. Even for moderately rough surfaces (Sq = 1.9 µm) release times are ≈ 20 s, much higher than acceptable times for industrial pick and place (duration can be <1 s, depending on the application). Since we used bipolar AC voltage, the slow release is arguably due to dry adhesion, which keeps the seal intact and hence holds a good vacuum. To overcome this limitation, we used a solenoid valve to vent the vacuum. To test the validity of our hypothesis, the valve was mounted below the surface of the substrate (Figure S2, Supporting Information). Using a valve for vacuum venting led to a drastic reduction in release time, from over 300 s (no valve, smooth PMMA) to <0.2 s (vacuum venting), even for the smooth (Sq = 0.5 µm) PMMA substrate. As the substrate roughness increases, the difference in release time with and without opening the valve decreases, but release times without opening the valve remain very long (20 s with Sq = 1.9 µm). Based on the results of these tests, we built an EA suction cup gripper with an integrated miniaturized valve inside its central pillar, as described in the following section.

### 2.5 Valve Integration and Pick and Place Demo

**Figure 5:**
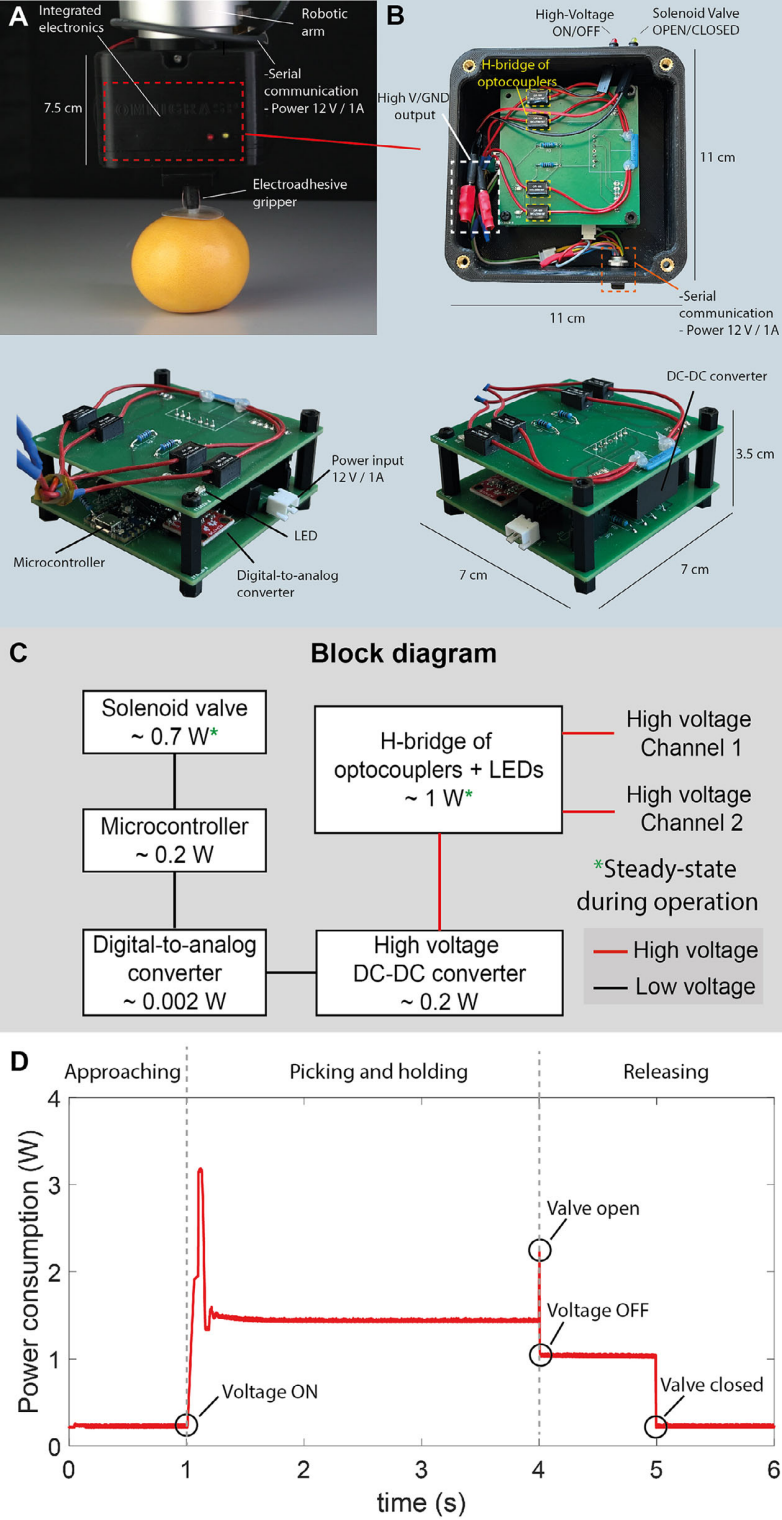
Compact high voltage power supply and its power consumption. Photos of the compact high‐voltage power supply used to control the EA suction cup for pick and place operations. A) The electronic box can be easily connected to commercial robotics arms. B) It consists of a 10 kV DC/DC converter, connected to an H‐bridge of optocouplers, which are controlled by an Arduino microcontroller. C) Block diagram of circuit board components, their connections, and their steady‐state power consumption. D) Power consumption during the three phases of a typical pick and place task.

Having shown that vacuum venting is a simple and effective solution to achieve fast release, we modified the design of the EA suction cups to integrate a miniature solenoid valve in the central pillar of the structure (Figure 2A). We also developed a compact high‐voltage power supply (**Figure** **5**A,B) to control both the high voltage to the EA electrodes and the low voltage to the solenoid valve. The power supply can be connected to and powered by commercial robotic arms, enabling robotic pick‐and‐place applications.

**Figure 6:**
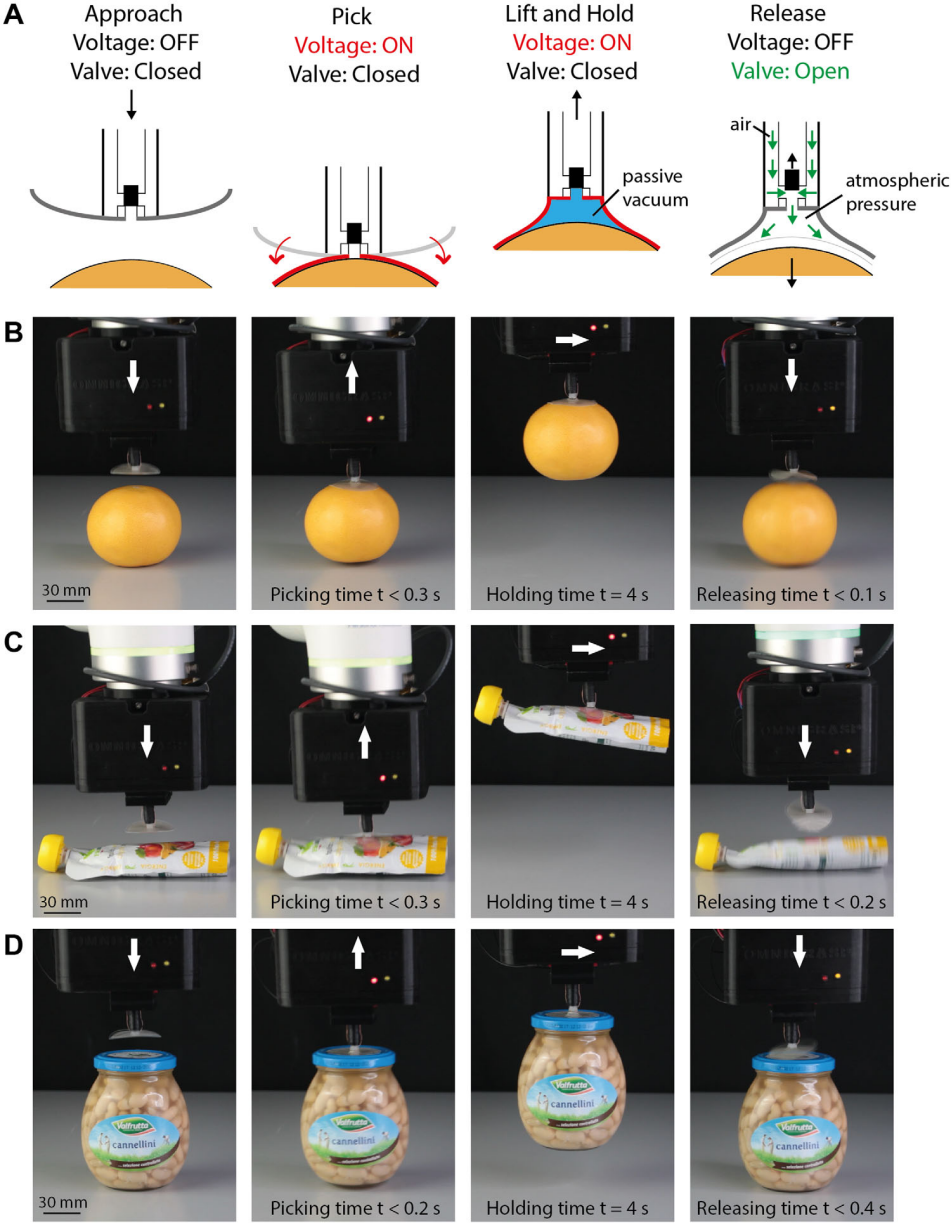
Picking and placing objects using an ElectroAdhesive suction cup. A) summary of the operating principle. B–D) Snapshots extracted from Movie S2 (Supporting Information), for three objects (orange, soft pouch, jar). The soft EA suction cup successfully holds and rapidly releases objects of different sizes, materials, and shapes.

We tested the performance of the gripper (same PDMS membrane used in the experimental characterization, diameter 4.3 cm) in the pick and place of a wide variety of real objects of different sizes, shapes, materials, and weights. **Figure** **6**A and Movie S1 (Supporting Information) show a pick and place cycle. (Step 1, Approach) the EA suction cup approaches the objects from the top until contact. (Step 2, Pick) the voltage is turned on, and the thin membrane very rapidly (50 ms) conforms to the object surface. (Step 3, Lift and Hold) the object is then lifted and moved to the target location. During these three steps, the valve remains closed to hold passive vacuum in the deformed membrane. (Step 4, Release) once the target location is reached, the voltage is turned off, and the valve is simultaneously opened. The objects are released in <0.4 s.

Movie S3 (Supporting Information) compares the release of several objects, comparing the release time when the valve remains closed to the one when it is opened, showing that vacuum venting greatly reduces the release time. Table S1 (Supporting Information) provides a comparison of release times with and without the activation of the valve. For most of the tested objects, release times are above 10 s with voltage off and the valve closed. Vacuum venting with the integrated miniaturized valve reduces release time from about 10 s to < 0.25 s, enabling fast release of a wide variety of objects.

**Figure 7:**
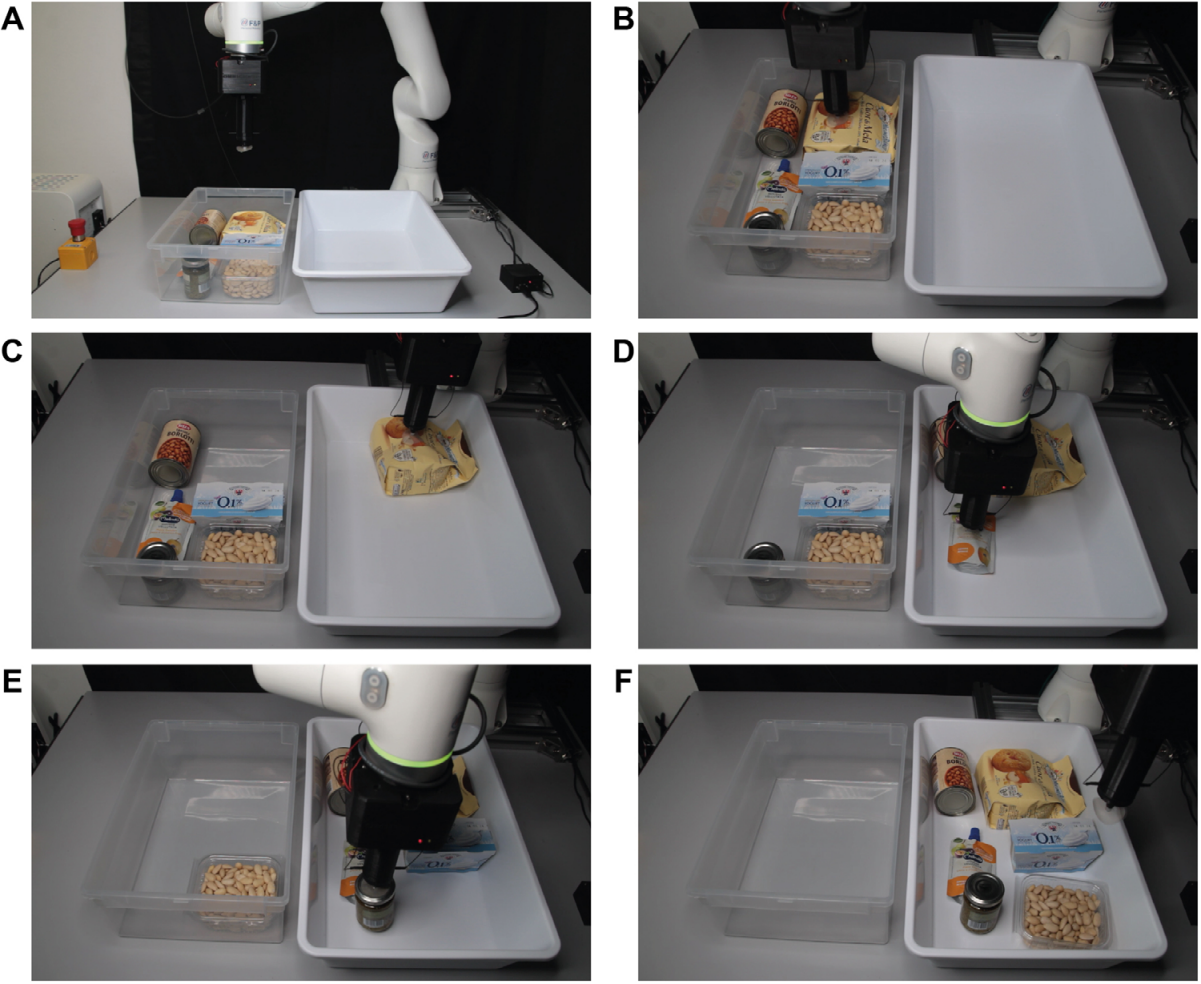
Bin picking demonstration. Images extracted from Movie S4 (Supporting Information). A) The demo starts with the six objects placed in the transparent box on the left. B–E) The suction cup design allows the ElectroAdhesive membrane to pick objects directly from the top. This is essential in scenarios in which lateral space is limited, and ElectroAdhesive grippers with fingered design would not have access to lateral surfaces to grasp the object. F) The demo ends with all the objects placed in the white tray.

Finally, we demonstrate the use of EA suction cups in a typical bin picking scenario (**Figure** **7**; Movie S4, Supporting Information). Bin picking is common in industry but cannot be accomplished by fingered EA grippers that need to grasp objects from the sides. EA suction cups grasp even heavy objects directly from the top, making bin picking accessible to EA grippers. In this demonstration, we show the successful picking and placing of six objects of different shapes, materials, weights, and sizes from one box to a second box.

### 2.6 Power Consumption

EA grippers have much lower power consumption than pneumatic systems relying on vacuum pumps or ejectors. In this section, we compare the power consumption of standard vacuum suction cups and our EA suction cups in typical pick‐and‐place scenarios. Estimating power consumption of vacuum suction cups is not trivial, as it is influenced by several factors including air leakage through seals, size and length of the connectors and couplings, suction cup volume, cycle time, and the chosen vacuum ejector. We did not include vacuum pumps in the estimation because they are not commonly used in pick‐and‐place applications.^[^ ^4^ ^]^ To make a conservative estimation, we assumed no leakage in the vacuum system, representing an ideal scenario for vacuum suction cups. We based our example on components and timing from a technical document by Festo SE & Co. KG “Basic principles of vacuum technology, brief overview”.^[^ ^4^ ^]^ We consider a pick‐and‐place cycle time of 4 s (the same cycle time that we used in the demo shown in Movie S2, Supporting Information) for an object of mass 1 kg with a smooth surface.

A cycle can be subdivided into four intervals: pick/evacuation (time t_E_), handling (time t_1_)_,_ release (time t_R_), and return (time t_2_). To provide a more detailed comparison, we considered two types of vacuum generators both with evacuation times <0.4 s: the VADMI–45 (0.26 s) and VN–07–H (0.12 s). The VADMI vacuum generators have an embedded non‐return valve, maintaining vacuum after the generator is switched off, leading to lower power consumption compared to the VN vacuum generators, which need to remain on during both the evacuation and holding times. Vacuum generators with energy‐saving functions are commonly used in pick and place of very smooth objects for which air leakage during holding time is negligible. They do not perform well on moderately rough and porous objects. We calculated the power consumption for each vacuum generator based on air consumption data from the ejectors manufacturer. The ideal power consumption (without air leakage) is ≈4.5 W for the VADMI‐45 (smooth objects only) and 68 W for the VN‐07‐H (moderately rough and porous objects), with actual consumption higher due to air leakage (see Supporting Information for detailed calculations). For active vacuum systems, power consumption scales linearly with the number of suction cups in operation on a line and with the number of cycles per hour. For a line with 10 suction cups and 60 cycles/minute (on the low side for an industrial assembling line) we reach 180 W with VADMI‐45 and 2.7 kW with the VN‐07‐H. For EA suction cups, the measured power consumption with our current lab‐grade power supply is below 1.5 W in steady‐state during pick and place cycles (Figure 5). Most of the power consumption comes from the H‐bridge optocouplers, the microcontroller, and the DC‐DC converter (Figure 5C). The total power consumed by our power supply during a typical pick and place cycle is plotted in Figure 5D.

The steady‐state power consumption is 1.5 W when holding the object. Short peaks of a maximum of 3 W (duration < 0.1 s) occur when turning on the DC‐DC converter and when opening the valves. One of our power supplies can drive >10 EA suction cups. It takes <1 ms to charge one EA suction cup, considering that the capacitance is ≈20 pF, the resistance of the electrical connections is ≈30 MΩ, and the maximum current output of the power supply is limited to 100 µA. Connecting 10 EA suction cups to one of our power supply leads to a charging time of about 10 ms. For 10 EA suction cups, one can use the same driving electronics of 1.5 W for EA, plus 10 valves, each using 0.7 W when opening, for a total of 1.5 W while holding and a short peak of 8.5 W when the valves are opened (object release). A set of 10 EA suction cups could be driven with total power consumption under 10 W, <10% of the power required by 10 ideal leakage‐free vacuum cups driven by power‐saving vacuum generators.

## 3 Discussion

This work reports electroadhesion (EA) suction cups: a class of EA soft grippers addressing the previously unsolved challenge of grasping objects from the top using Electroadhesion. EA suction cups have a lifting force of over 10 N, compared to 10 mN for previously reported fingered EA grippers when lifting from the top.

This increase in performance is achieved through two main innovations: 1) the EA membrane is unconstrained and free to conform to the shape of the object and 2) we engineered the detachment process when lifting the object to maximize the EA peeling forces and create EA‐induced vacuum between the gripper and the object. We use circular EA membranes that are thin, soft, and with free boundaries. This design leaves the membrane free to deform under EA forces, conforming to the shape of the object when the high voltage is applied. Large area contact is established with flat and non‐flat objects and with surface roughness tested up to 1.9 µm. To produce high lifting forces, we studied and engineered the detachment process of the membrane from the object. We used a combination of geometry and materials that make the membrane detach from the center, where the pillar is connected, with a circular and expanding peeling boundary. The peeling angle is kept low by the shape of the detached portion of the membrane and a vacuum‐filled chamber forms underneath the central pillar.

We tested EA suction cups of different sizes and made of different materials. We obtained lifting forces up to 18 N with a 5.3 cm diameter membrane. Grasping and release times are below 400 ms. Fast release is enabled by a miniature solenoid valve that quickly vents the EA‐induced vacuum. We report successful grasping of everyday objects of different shapes and materials in a bin picking scenario (Movie S4, Supporting Information). The EA suction cups we tested showed very good grasping performance for object sizes from 5 to 20 cm. For smaller objects, the pillar width would cover a large portion of the object, leaving only a small space for the EA membrane to adhere on the object. A smaller membrane with a redesigned thinner pillar would give better performance for small objects. Larger and heavier objects can be successfully grasped by using sets of EA suction cups in parallel.

Grasping performance is influenced by object roughness. We tested controlled surfaces and objects with roughness values in the range Sq = 0.5–1.9 µm, observing reliable grasping performances. Higher roughness values have a combined effect of reducing the EA forces and increasing the air leakage between the vacuum chamber and outside air, both due to reduced area contact between the gripper and the object. The result is that grasping performance is globally reduced. There exists a wide range of roughness values above Sq = 1.9 µm where objects can still be grasped but with reduced performance and reliability. For such higher roughness values, the grasping performance needs to be assessed on an object‐by‐object basis. Very high roughness objects, such as a kiwi fruit or a wool sweater generate very low forces, so cannot be grasped with the current gripper embodiment. Finally, we tested an EA suction cup for over 11 000 pick‐and‐place cycles (>15 h of continuous operation) and observed no degradation (Movie S5, Supporting Information).

Some challenges need to be overcome for the industrial adoption of EA suction cups. Their grasping performance can be affected by dust and debris accumulation on the EA membrane after a large number of cycles. We observed a decrease in grasping performance after 9000 cycles on a paper object. Cleaning by wiping with alcohol solved the issue. After this cleaning step, the grasping performance returned to its initial level (Movie S5, Supporting Information). For industrial operations, one can imagine using automated cleaning cycles with a cleaning frequency depending on the material and cleanliness of the grasped object.

Temperature and humidity in common laboratory conditions (20–25 °C, 30–50% RH) do not influence gripper performance. We anticipate temperature to have no significant influence in a wider range (PDMS is stable in a range from −50 to 200 °C). Lower humidity would also have no effect, while much higher humidity (e.g., 90% RH) can lead to reduced lifetime of the EA membrane due to reduced dielectric breakdown of PDMS.^[^ ^41^ ^]^ Condensing humidity can reduce EA performance if water is formed between the gripper and the object. Applications requiring uncommon ambient conditions will need dedicated performance evaluation at higher TRL.

Some challenges arise from the large deformations in the thin and soft EA membrane. While softness is required for creating EA‐induced vacuum, it leads to oscillations when moving heavy objects at high speeds. These oscillations can lead to premature detachment or inaccurate positioning. Oscillations can be reduced by grasping larger and heavier objects with more than one suction cup, as multiple (non‐aligned) grasping points offer higher stability by greatly increasing rotation stiffness. Such a configuration can lead to accurate pick‐and‐place, and enables lifting heavier loads while enabling object rotation. Advanced motion planning is, however, required to place each suction cup in contact with the object.

EA suction cups can replace conventional suction cups in a wide range of pick‐and‐place operations. Similar to vacuum cups, EA suction cups have a small footprint and can grasp objects from the top. In addition, they present unique advantages: 1) significantly lower power consumption (we estimated <10% of power required by suction cups using active vacuum, when driving an array of 10 grippers), 2) silent operation, 3) compact and portable driving system, 3) fully electrical operation, with no flow of air around the object. However, there are classes of objects that could not be successfully grasped using EA suction cups. Highly porous objects show very low lifting forces since the EA‐induced vacuum cannot form and the low‐area contact leads to small EA forces. Objects with moderate to large Gaussian curvature are also challenging to grasp since with the current design the membrane would need large in‐plane deformations to conform to their shape. This challenge could be tackled by a design that reduces the stretching required to conform to a given curvature.

The design and results presented in this article report that EA suction cups can be used as an alternative to conventional vacuum cups in bin picking pick and place operations. Further studies on materials, geometry, contact mechanics, and electrostatic actuation will tackle some of the limitations of the current design and lead to better‐performing and more mature EA suction cups offering a silent, low‐power, and highly compact alternative to the widely used suction cup grippers.

## 4 Experimental Section

### Electroadhesive Membrane Fabrication

Two types of EA suction cups were fabricated using either a soft and stretchable bottom layer or a stiff and flexible bottom layer. For stretchable membranes: A 50 µm thick PDMS layer (Waker Elastosil 2030) was cut and placed on the plate of a manual screen printer (Charmhigh 3040 High Precision Manual Solder Paste Printer) and used as the bottom layer. The interdigitated electrodes were screen printed using a silicone‐based electrically conductive silver ink on the PDMS substrate. The ink was cured at 150 °C for 1 h. After ink curing, a 300 µm thick layer of Syligard 184 (Dow Corning) was blade‐casted on the PDMS substrate and then cured at 80 °C for 2 h. Finally, the circular shape of the membrane was obtained using a laser cutter (Trotec Speedy 300) leaving a margin of 2 mm from the electrode pattern to avoid the risk of damaging the electrodes in the proximity of the laser cut. The mesh was designed to screen‐print electrode patterns with different diameters (4.9, 3.9, and 2.9 cm). The electrical connections were obtained by punching holes in the middle of the membrane and filling them with silver epoxy, to ensure electrical contact with the screen‐printed ink inside the membrane. Thin copper wires (0.23 mm) were then used to connect the membrane electrodes to the power supply.

The procedure to fabricate flexible rather than stretchable membranes used to quantify the effect of membrane stiffness in Figure 4C is the same as the one described above for stretchable membranes, but instead of using PDMS as the bottom layer, we used thin sheets of Mylar (0.23 mm thickness E = elastic modulus 4.1 GPa).

### Electroadhesive Membrane Lifting Performance Characterization

The lifting performance of the electroadhesive membranes was characterized using the setup shown in Figure S1 (Supporting Information). The setup consists of the electroadhesive membrane, attached to a 3D‐printed soft pillar made of Polylactic Acid (PLA), using silicone glue (Sil‐poxy, Smooth‐on), which was then connected with screws to an Instron mechanical tester (INSTRON 3340 Single Column Universal Testing System) equipped with a 50 N load cell (Instron 2519–50N). PMMA rigid plates (5 mm thickness) were used as substrates in contact with the electroadhesive membranes. Before each test, both the electroadhesive membrane and the PMMA substrate were cleaned with isopropyl alcohol, to remove dust and possible residual surface charge.^[^ ^42^ ^]^ At the beginning of the tests, the electroadhesive membrane was moved downward until it contacted the PMMA substrate reaching a compressive force of 0.2 N. This procedure ensured that the center of the membrane was in full contact with the substrate. Then, a voltage of 3 kV DC was applied, and the membrane zips on the substrate. The pillar was then raised at a constant velocity of 0.2 mm s^−1^ and the lifting force was measured at 100 ms time intervals.

To evaluate the effect of roughness on lifting performance, three PMMA substrates were used: one smooth as purchased, one polished with sandpaper with grit size 80J, and one with grit size 320J. The roughness of the three substrates was measured using a confocal laser microscope (Keyence VK X‐1000). To increase contrast on the transparent PMMA surface, a thin layer of gold (≈10 nm) was sputtered on the substrate. The results are shown in Figure 3B.

Each test was repeated three times. For roughness and substrate with and without hole experiments, curves represent mean values, and the shaded area represents the standard deviation. For diameter, voltage, and stretchable versus flexible membrane experiments, all three repetitions are plotted.

### Electroadhesive Membrane Release Time Characterization

Release time tests were conducted using the setup shown in Figure S2 (Supporting Information). The setup was identical to the one used for lifting performance characterization (see previous paragraph) except for the substrate. Initially, the electroadhesive membrane was moved downward toward the box until a compression force of 0.2 N was reached. In this initial phase, the voltage was off. Then a bipolar voltage of 3 kV at 5 Hz was applied. The box was then raised at a vertical speed of 5 mm s^−1^ until a vertical displacement of 12 mm was reached. Release times were then evaluated in two conditions: i) the applied voltage was turned off after 4 s and the valve was kept closed, ii) the applied voltage was turned off after 4 s and the valve was simultaneously opened. When the valve was closed, the volume inside the chamber formed by the EA membrane deformation was isolated from the atmosphere, and a passive vacuum was created due to the membrane deformation caused by the weight of the box (Figure S2Bi). When the valve was open, the deformed chamber was no longer isolated, atmospheric pressure entered inside the deformed chamber, the passive vacuum vanished very quickly, and the box was easily released (Figure S2B(ii)). Release time is computed from the video of the experiments (recorded at 24 frames per second). It starts when the voltage is deactivated and ends either at the moment in which the box detaches from the EA membrane or after 300 s. Tests were repeated three times for each substrate roughness. The mean and standard deviation are reported in Table 2.

### Electroadhesive Membrane Vacuum Pressure Characterization

The vacuum pressure inside the conical deformed chamber of EA suction cups generated during membrane lifting was characterized using the setup shown in Figure S3 (Supporting Information). The setup was identical to the one used for lifting performance characterization with the addition of a vacuum pressure sensor (NXP Semiconductors MPXH6115A6T1) below the PMMA rigid plate, as shown in the schematic view of Figure S3 (Supporting Information). At the beginning of the tests, the electroadhesive membrane was moved downward until it contacted the PMMA substrate, reaching a compressive force of 0.2 N. This procedure ensures that the center of the membrane is in full contact with the substrate. Then, a voltage of 3 kV DC was applied, and the membrane zips on the substrate. The pillar was then raised at a constant velocity of 1 mm s^−1^ until a vertical displacement of 4 mm displacement was reached. During lifting, the force from the load cell, vacuum pressure, and detached area were measured. The detached area during lifting was measured optically using motion‐tracking software (kinovea.org). A comparison of the force measured by the load cell and the force due to passive vacuum, obtained by multiplying the vacuum pressure by the detached area, is shown in Figure S4A (Supporting Information). Tests were repeated three times. The mean and standard deviation are reported in Figure S4 (Supporting Information). The maximum vacuum pressure measured at 4 mm of vertical displacement was 62 kPa (Figure S4D, Supporting Information), which is <101 kPa as computed for the experiments shown in Figure 2A for the case of the smooth substrate. This difference was probably due to the presence of a dead volume introduced by the pressure sensor. The initial volume of air underneath the membrane was significantly higher in the presence of the vacuum pressure sensor. Since vacuum pressure was proportional to volume variation of the deformed chamber during lifting, starting with a higher initial volume leads to a lower vacuum pressure at the same vertical displacement. While the presence of the pressure sensor influences the vacuum pressure, it did not influence the comparison between the force measured by the load cell and the one due to passive vacuum, which was the goal of this experiment.

## Conflict of Interest

F.C. and V.C. declare financial interest in the form of a patent application.

## Author Contributions

F.C. and V.C. formulated the concept; F.C. fabricated the devices, carried out the experiments, analyzed the data, and created the figures and videos, with the supervision from H.S. and V.C.; F.C. prepared the original draft of the manuscript; F.C., H.S., and V.C. reviewed and edited the manuscript.

## Supporting information



Supporting Information

Supplemental Movie 1

Supplemental Movie 2

Supplemental Movie 3

Supplemental Movie 4

Supplemental Movie 5

## Data Availability

The data that support the findings of this study are openly available in Zenodo at 10.5281/zenodo.13929745, reference number 1.
