# Supplementary material for: Electroadhesion Suction Cups
Source: Adv Mater. 2025 Apr 24;37(27):2420231. doi: 10.1002/adma.202420231 (PMC12243719; doi:10.1002/adma.202420231)
Supplement: Supplementary file 1 — Supporting Information [file ADMA-37-2420231-s002.docx]

Supplementary Materials

**Power consumption and comparison with conventional vacuum grippers**

In the following computation, we will assume no leakage in the vacuum system, which represents an idealized scenario for vacuum suction cups (in reality leaks in the line will significantly increase the power consumption). We consider a pick-and-place cycle time of four seconds (for an object of mass 1 kg with a smooth surface. A cycle can be subdivided into four time intervals: pick (evacuation) time t_E,_ handling time t_1,_ release time t_R_, and return time t_2._ The evacuation time is the time required to reach a certain vacuum level in the total volume of the vacuum system (V_t_ = V_suction cup_ + V_holder_ + V_tube_). We chose a suction cup from the Festo catalogue with a breakaway force exceeding 10 N (ESS-20-BN) with a volume of 1.6 cm^3^ (V_suction cup_). From the same catalogue, we chose a suction cup holder (ESH-HD-4-QS) with a volume of 0.7 cm^3^ (V_holder_). To connect the vacuum generator to the suction cups we chose a tube of length L = 1 m with an external/internal diameter of 6/4 mm, for a volume of 12.6 cm^3^. The total volume of the vacuum system is therefore V_t_ = 15 cm^3^. The choice of the vacuum generator depends on its evacuation time t_E_. We chose two vacuum generators with evacuation times below 0.4 s: the VADMI–45 and VN–07–H, with evacuation times of 0.26 s and 0.12 s, respectively. The times are computed from the datasheet of the vacuum generator to reach a vacuum pressure of 0.7 bar in the total volume of the vacuum system V_t_). The power consumption of the vacuum ejectors depends on the air consumption Q expressed in liters per minute (l/min) of compressed air (typically at 7 bar) needed to generate the required vacuum pressure in the system. The air consumption per operation cycle $Q_{z}$ is given by the formula $Q_{z}=t_{ON}Q$. The total time in which the vacuum generator remains on is $t_{ON}= t_{E}$ for the vacuum generator with energy savings and $t_{ON}= t_{E}+ t_{1}$ without energy savings. Considering a handling time $t_{1}=1.5 s$ and air consumption from the datasheets (VADMI-45:$Q=\frac{11}{60} l/s$) and (VN-07-H:$Q =\frac{28}{60} l/s$), the air consumption per operation cycle is $Q_{z}=0.05 l$for VADMI-45 and $Q_{z}=0.76 l$ for VN-07-H. The energy spent to generate compressed air at 7 bar is approximately E_CA_ = 0.1 kWh/m^3^. The energy spent per cycle is given by E_CYCLE_ = E_CA_$Q_{z}$, which gives 0.005 Wh for VADMI-45 and 0.076 Wh for VN-07-H. With a cycle time of 4 s there are 900 cycles per hour ($N_{c}$). Therefore, the power consumption is given by the formula $P= E_{CYCLE}N_{c}$. Substituting the values of $E_{CYCLE}$ of the vacuum generators we obtain a power consumption of ~ 68 W for VN-07-H and ~ 4.5 W for VADMI-45.

**
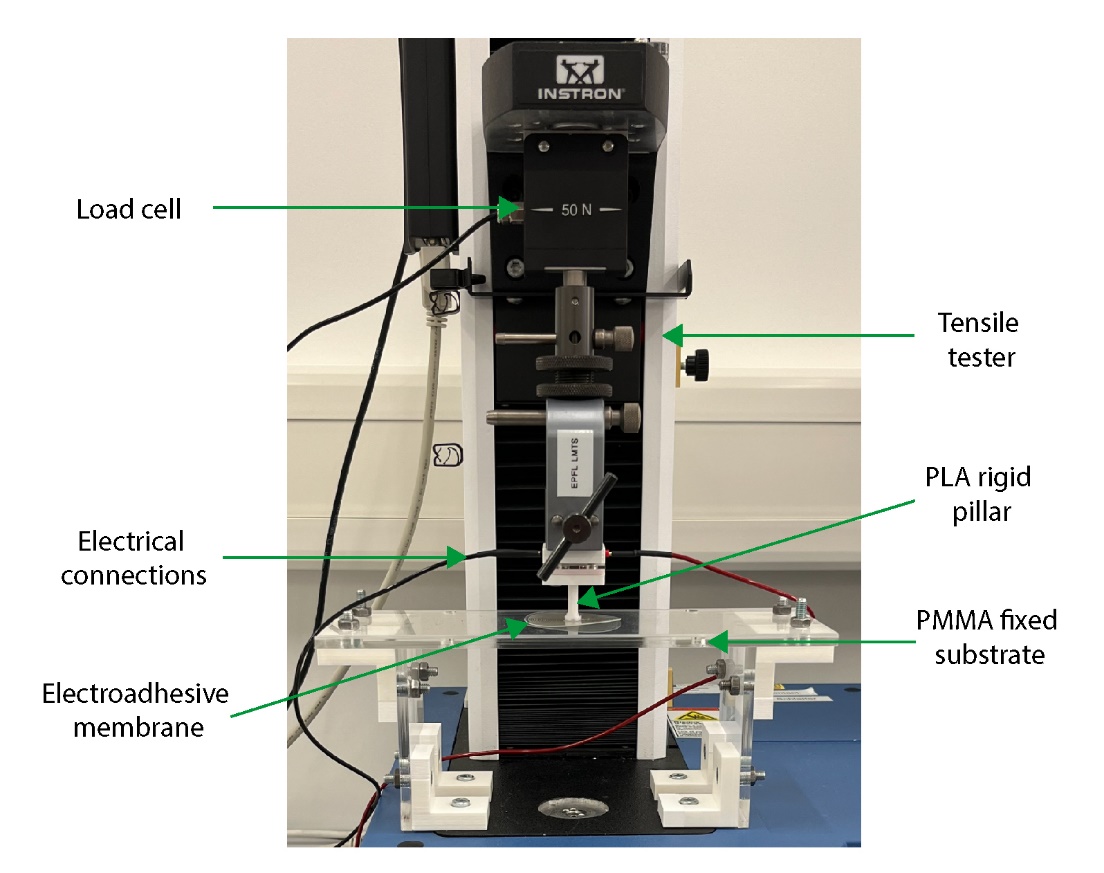
**

Fig. S1. Experimental set-up used to characterize lifting performance of the electroadhesive membranes.


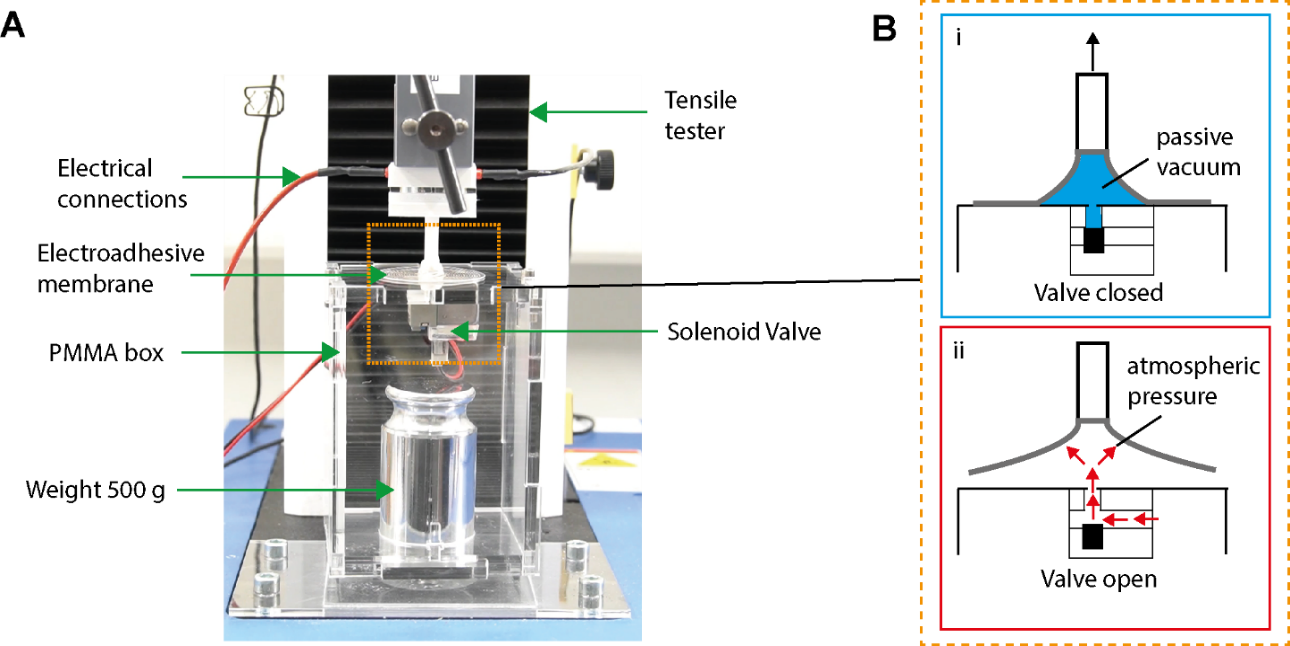


Fig. S2. Experimental set-up used to characterize release time on different substrate roughness with and without the activation of a solenoid valve. (A) Visualization of the experimental set-up used to characterize release time. (B) Schematic of the vacuum pressure inside the deformed chamber when the valve is closed (i) and the airflow at atmospheric pressure and the subsequent loss of vacuum pressure when the valve is open (ii).


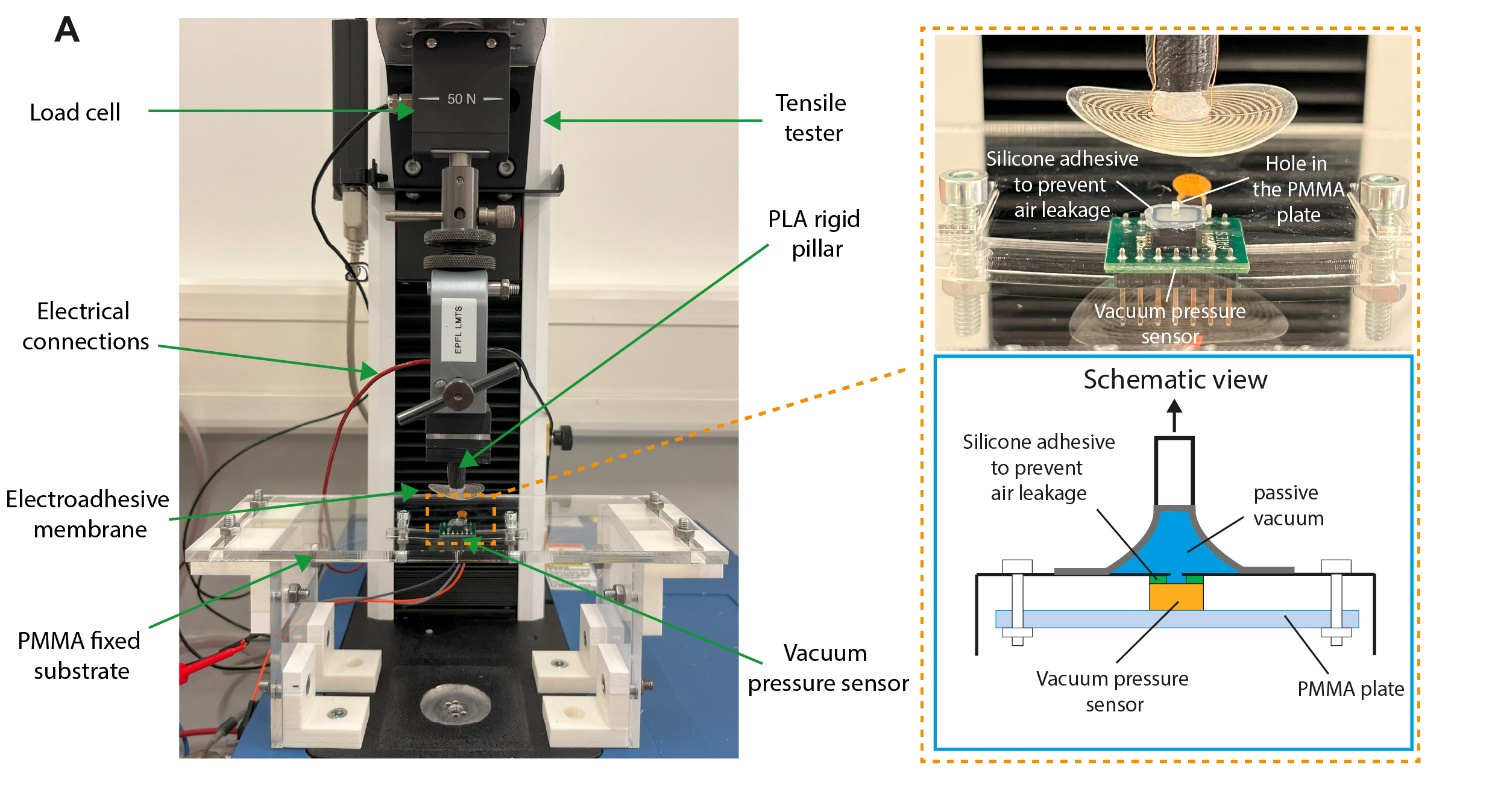
**Fig. S3. Experimental set-up to measure the vacuum inside the detached conical chamber when raising the EA membrane. The pressure’s sensor small form factor allows for a very small additional dead volume.**


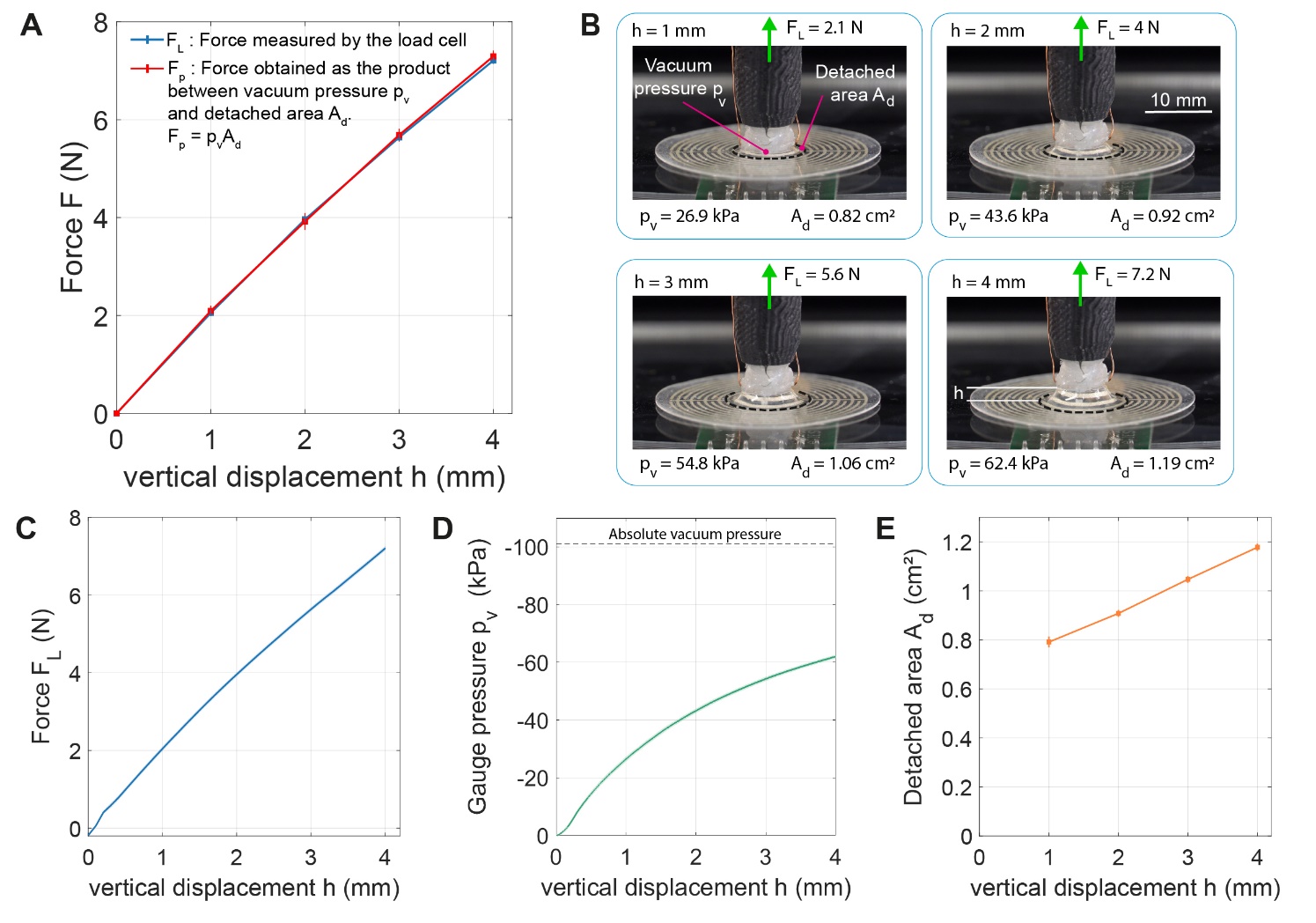


**Fig. S4. Measuring EA-induced passive vacuum pressure during EA membrane lifting.** All data is for an EA suction cup of diameter 3.3 cm and with an applied voltage of 3kV DC. The main goal of this experiment is to determine the relative importance of EA force vs vacuum force.
(**A**) Comparison of the force directly measured by the load cell F_L_ (blue curve) and the force F_p_ (red curve) computed by multiplying the measured gauge pressure p_v_ by the detached area A_d_, as a function of vertical displacement of the central attachment. The excellent overlap between the two curves demonstrates that the high lifting forces are almost entirely due to passive vacuum pressure inside the deformed conical chamber, while electroadhesion lifting force is negligible. Electroadhesion pressure, however, plays an essential role in ensuring an airtight seal, needed for the passive vacuum. (**B**) Images of the EA membrane during lifting at different vertical displacements (1 mm, 2 mm, 3 mm, and 4 mm), along with the measured vacuum pressure and detached area. The bottom three plots are: (**C**) Force measured by the load cell, (**D**) gauge pressure, and (**E**) detached area as a function of vertical displacement. This data is used to generate the plot in (**A**).

|  | Material | Release time (s): Voltage OFF  Valve Closed | Release time (s):  Voltage OFF  Valve Open |
| --- | --- | --- | --- |
| Juice pouch  (130 g) | C/LDPE 90 Plastics/Aluminium | > 10 s | < 0.16 s |
| Biscuits cylinder (190 g) | C/PAP 84 Cardboard/Plastic/Aluminium | **<** 9 s | **<** 0.2 s |
| Grapefruit  (230 g) | Fruit | > 10 s | **<** 0.25 s |
| Cylindrical can  (350 g) | 41 ALU  Aluminium | > 10 s | **<** 0.13 s |
| Biscuits bag  (370 g) | C/PAP 81  Paper/Plastic | < 2 s | **<** 0.09 s |
| Eggplant  (480 g) | Vegetable | > 10 s | **<** 0.09 s |
| Pasta paper box  (512 g) | PAP 21  Non-corrugated fiberboard | < 0.09 s | **<** 0.09 s |
| Grape basket  (530 g) | PET 1  (Polyethylene terephthalate) | > 10 s | < 0.09 s |
| Tomato sauce bottle (550 g) | 70 GL  Clear Glass | > 10 s | **<** 0.13 s |
| Beans can  (570 g) | Fe 40  Steel | > 10 s | **<** 0.3 s |

Table S1. Release time comparison with the valve open and closed on different objects. Release time is measured from video recordings, values are mean, N = 3 trials for each object, with an uncertainty of ±0.04 s due to the frame rate of 24 fps.


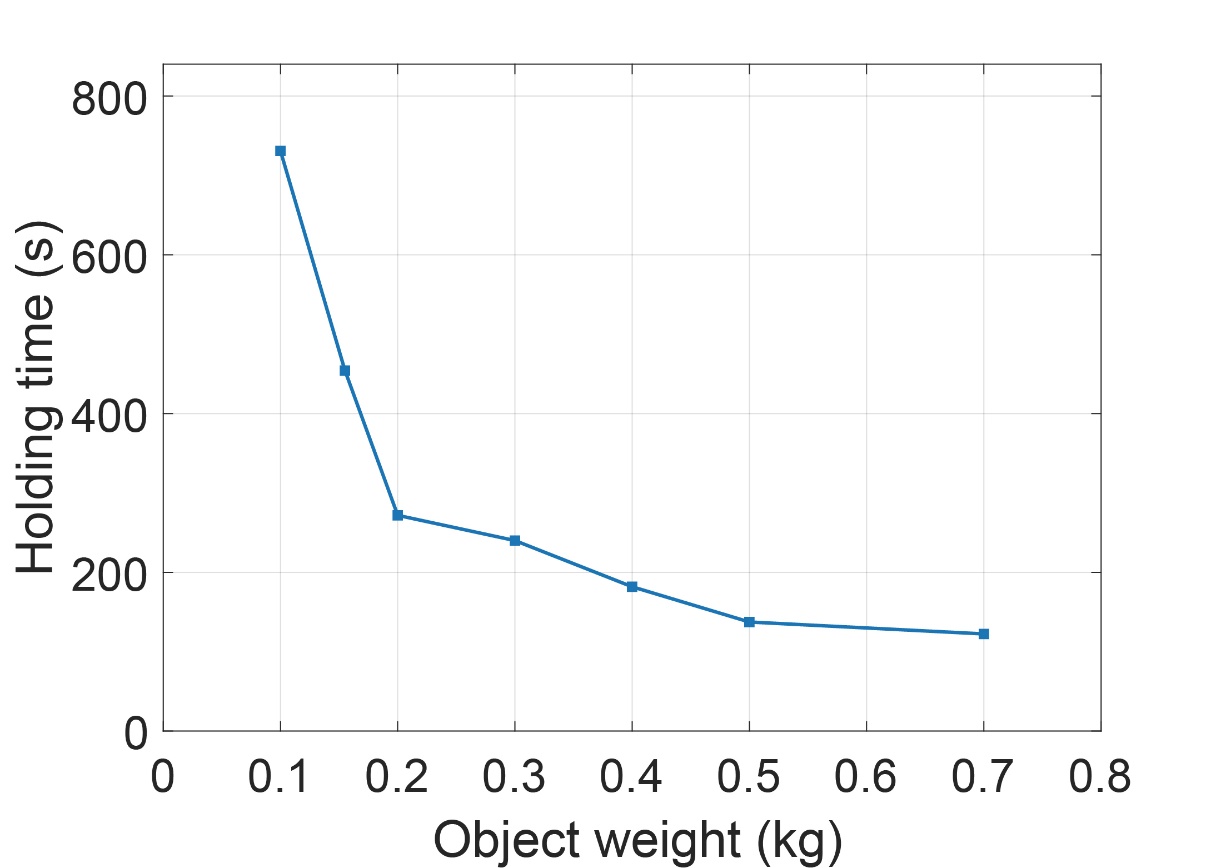


**Fig. S5. Maximum holding time when lifting a box with substrate roughness Sq = 1.5 µm loaded with different weights**.

**Movie S1**. **Electroadhesion suction cups.** Summary video of device performance and operating principle.

Movie S2. Electroadhesion suction cups. Pick and place of different objects. The electroadhesion suction cup and high-voltage power supply are mounted on the wrist of a robotic arm, demonstrating successful pick-and-place operations with various objects. The audio in the video highlights the silent operation of the EA suction cups.

Movie S3. Release time comparison: Voltage off + valve closed, Voltage off + valve open. A demonstration of the different release times with and without using the valve.

Movie S4. Bin picking of different objects with an electroadhesion suction cup. A demonstration of a bin picking scenario performed by the electroadhesion suction cup. The demo starts with six objects placed in a transparent box on the left. The electroadhesion membrane picks up the objects from the top and places them in another tray on the right.

Movie S5. Lifetime evaluation of EA suction cups through continuous pick and place operations. The lifetime of electroadhesion suction cups is evaluated by performing continuous pick and place of a yogurt case.
